# Supplementary material for: Detection of left ventricular systolic dysfunction from single-lead electrocardiography adapted for portable and wearable devices
Source: NPJ Digit Med. 2023 Jul 11;6:124. doi: 10.1038/s41746-023-00869-w (PMC10336107; doi:10.1038/s41746-023-00869-w)

ONLINE SUPPLEMENT

Khunte A, Khera R, et al. Detection of Left Ventricular Systolic Dysfunction from Single-Lead Electrocardiography Adapted for Portable and Wearable Devices

TABLE OF CONTENTS

SUPPLEMENTARY TABLES.. 2

Supplementary Table 1: Patient-level baseline characteristics of study population........ 2

Supplementary Table 2: Performance of noise-adapted and standard model on ECGs augmented with individual noise signatures... 3

Supplementary Table 3: Performance of noise-adapted and standard model on ECGs augmented with multiple noise signatures... 4

SUPPLEMENTARY FIGURES.. 5

Supplementary Figure 1: Flow chart of study cohort and analysis.................................. 5

Supplementary Figure 2: Model architecture used for model development.................... 6

SUPPLEMENTARY TABLES

Table S1. Patient-level baseline characteristics of study population. Data presented median [IQR] for age and number (percent) for other variables.

Characteristic	Total (N = 116,210)
Sex	
Female	50,776 (43.7%)
Male	53,271 (45.8%)
Unknown	12,149 (10.5%)
Age (years)	68 [56-78]
Race	
Hispanic	9,349 (8.0%)
White	75,928 (65.3%)
Black	14,000 (12.0%)
Other	16,843 (14.5%)

Table S2. Performance of noise-adapted and standard model on different noise types at each signal-to-noise ratio on the held-out test set. Abbreviations: SNR, signal-to-noise ratio; PPV, positive predictive value; NPV, negative predictive value; AUROC, area under receiver operating characteristic curve; AUPRC, area under precision recall curve.

Model	Noise	SNR	PPV	NPV	Specificity	Sensitivity	AUROC (95% CI)	AUPRC
Noise-Adapted	None	N/A	0.203	0.990	0.676	0.924	0.896 (0.886-0.905)	0.455
	Portable ECG	0.50	0.152	0.993	0.523	0.956	0.871 (0.861-0.882)	0.392
	Portable ECG	0.75	0.165	0.992	0.569	0.951	0.879 (0.868-0.889)	0.411
	Portable ECG	1.00	0.172	0.992	0.592	0.946	0.883 (0.872-0.893)	0.421
	Portable ECG	1.25	0.177	0.992	0.607	0.945	0.885 (0.875-0.895)	0.428
	Portable ECG	1.50	0.181	0.992	0.619	0.942	0.887 (0.877-0.897)	0.433
	Portable ECG	1.75	0.185	0.992	0.628	0.942	0.888 (0.878-0.898)	0.436
	Portable ECG	2.00	0.188	0.992	0.636	0.940	0.889 (0.880-0.899)	0.439
	Electrode Motion	0.50	0.126	0.993	0.395	0.971	0.858 (0.846-0.869)	0.361
	Electrode Motion	0.75	0.138	0.994	0.459	0.968	0.870 (0.859-0.881)	0.388
	Electrode Motion	1.00	0.147	0.993	0.501	0.961	0.876 (0.865-0.886)	0.402
	Electrode Motion	1.25	0.154	0.992	0.531	0.955	0.879 (0.869-0.889)	0.410
	Electrode Motion	1.50	0.160	0.992	0.553	0.953	0.881 (0.871-0.892)	0.416
	Electrode Motion	1.75	0.165	0.992	0.569	0.950	0.883 (0.873-0.893)	0.422
	Electrode Motion	2.00	0.167	0.991	0.579	0.945	0.885 (0.875-0.895)	0.426
	Muscle Artifact	0.50	0.175	0.989	0.608	0.927	0.871 (0.861-0.882)	0.389
	Muscle Artifact	0.75	0.182	0.990	0.626	0.930	0.879 (0.869-0.890)	0.415
	Muscle Artifact	1.00	0.186	0.990	0.636	0.931	0.884 (0.873-0.894)	0.429
	Muscle Artifact	1.25	0.190	0.991	0.643	0.934	0.886 (0.877-0.896)	0.437
	Muscle Artifact	1.50	0.192	0.991	0.649	0.932	0.888 (0.879-0.898)	0.444
	Muscle Artifact	1.75	0.194	0.991	0.652	0.934	0.890 (0.880-0.899)	0.448
	Muscle Artifact	2.00	0.194	0.990	0.654	0.930	0.891 (0.881-0.900)	0.449
	Baseline Wander	0.50	0.186	0.990	0.634	0.932	0.883 (0.873-0.893)	0.427
	Baseline Wander	0.75	0.192	0.990	0.651	0.926	0.887 (0.877-0.897)	0.440
	Baseline Wander	1.00	0.195	0.990	0.658	0.926	0.889 (0.879-0.899)	0.448
	Baseline Wander	1.25	0.198	0.990	0.664	0.926	0.890 (0.881-0.900)	0.453
	Baseline Wander	1.50	0.199	0.990	0.666	0.928	0.891 (0.881-0.901)	0.455
	Baseline Wander	1.75	0.200	0.991	0.668	0.929	0.892 (0.882-0.902)	0.457
	Baseline Wander	2.00	0.201	0.991	0.669	0.929	0.892 (0.883-0.902)	0.457
Standard	None	N/A	0.207	0.988	0.688	0.910	0.895 (0.884-0.905)	0.475
	Portable ECG	0.50	0.091	0.981	0.132	0.972	0.723 (0.706-0.739)	0.200
	Portable ECG	0.75	0.096	0.987	0.182	0.973	0.763 (0.748-0.779)	0.242
	Portable ECG	1.00	0.102	0.990	0.233	0.974	0.788 (0.773-0.802)	0.270
	Portable ECG	1.25	0.108	0.990	0.287	0.969	0.805 (0.791-0.819)	0.291
	Portable ECG	1.50	0.114	0.990	0.331	0.964	0.818 (0.805-0.831)	0.306
	Portable ECG	1.75	0.120	0.991	0.369	0.961	0.827 (0.814-0.840)	0.318
	Portable ECG	2.00	0.125	0.991	0.398	0.958	0.834 (0.822-0.847)	0.329
	Electrode Motion	0.50	0.088	0.988	0.079	0.990	0.792 (0.779-0.806)	0.239
	Electrode Motion	0.75	0.101	0.991	0.219	0.978	0.815 (0.802-0.829)	0.264
	Electrode Motion	1.00	0.114	0.991	0.330	0.965	0.829 (0.817-0.842)	0.285
	Electrode Motion	1.25	0.127	0.990	0.414	0.954	0.839 (0.827-0.851)	0.301
	Electrode Motion	1.50	0.137	0.989	0.468	0.942	0.846 (0.834-0.858)	0.313
	Electrode Motion	1.75	0.145	0.989	0.508	0.934	0.851 (0.839-0.862)	0.324
	Electrode Motion	2.00	0.152	0.988	0.537	0.925	0.855 (0.843-0.866)	0.333
	Muscle Artifact	0.50	0.100	0.991	0.210	0.979	0.807 (0.795-0.820)	0.245
	Muscle Artifact	0.75	0.124	0.993	0.385	0.970	0.832 (0.820-0.844)	0.274
	Muscle Artifact	1.00	0.143	0.991	0.488	0.951	0.844 (0.833-0.855)	0.294
	Muscle Artifact	1.25	0.157	0.990	0.546	0.940	0.851 (0.840-0.863)	0.310
	Muscle Artifact	1.50	0.166	0.989	0.584	0.929	0.857 (0.846-0.868)	0.323
	Muscle Artifact	1.75	0.175	0.989	0.612	0.923	0.861 (0.850-0.872)	0.335
	Muscle Artifact	2.00	0.181	0.988	0.631	0.912	0.864 (0.853-0.875)	0.344
	Baseline Wander	0.50	0.095	0.993	0.158	0.987	0.802 (0.789-0.816)	0.236
	Baseline Wander	0.75	0.113	0.990	0.324	0.964	0.822 (0.810-0.835)	0.260
	Baseline Wander	1.00	0.130	0.989	0.433	0.946	0.833 (0.821-0.846)	0.277
	Baseline Wander	1.25	0.144	0.988	0.503	0.933	0.841 (0.829-0.853)	0.292
	Baseline Wander	1.50	0.154	0.987	0.546	0.923	0.847 (0.835-0.859)	0.303
	Baseline Wander	1.75	0.163	0.988	0.578	0.918	0.851 (0.840-0.863)	0.314
	Baseline Wander	2.00	0.169	0.986	0.599	0.908	0.855 (0.843-0.866)	0.322

Table S3. Performance of noise-adapted and standard model on ECGs augmented with multiple noise types simultaneously at each signal-to-noise ratio on the held-out test set. Abbreviations: SNR, signal-to-noise ratio; PPV, positive predictive value; NPV, negative predictive value; AUROC, area under receiver operating characteristic curve; AUPRC, area under precision recall curve.

Model	Noise	SNR	PPV	NPV	Specificity	Sensitivity	AUROC (95% CI)	AUPRC
Noise-Adapted	Portable ECG + Electrode Motion	0.500	0.994	0.437	0.969	0.856	0.856 (0.845-0.867)	0.345
	Portable ECG + Electrode Motion	0.750	0.993	0.518	0.960	0.869	0.869 (0.859-0.880)	0.375
	Portable ECG + Electrode Motion	1.000	0.992	0.557	0.953	0.876	0.876 (0.865-0.886)	0.391
	Portable ECG + Electrode Motion	1.250	0.992	0.581	0.947	0.879	0.879 (0.869-0.889)	0.399
	Portable ECG + Electrode Motion	1.500	0.991	0.597	0.941	0.881	0.881 (0.871-0.891)	0.406
	Portable ECG + Electrode Motion	1.750	0.991	0.612	0.941	0.883	0.883 (0.873-0.893)	0.412
	Portable ECG + Electrode Motion	2.000	0.991	0.622	0.939	0.885	0.885 (0.875-0.895)	0.416
	Portable ECG + Muscle Artifact	0.500	0.990	0.568	0.937	0.872	0.872 (0.861-0.882)	0.386
	Portable ECG + Muscle Artifact	0.750	0.991	0.611	0.935	0.880	0.880 (0.869-0.890)	0.407
	Portable ECG + Muscle Artifact	1.000	0.990	0.633	0.930	0.884	0.884 (0.874-0.894)	0.418
	Portable ECG + Muscle Artifact	1.250	0.990	0.649	0.927	0.886	0.886 (0.876-0.896)	0.425
	Portable ECG + Muscle Artifact	1.500	0.990	0.659	0.926	0.888	0.888 (0.878-0.897)	0.430
	Portable ECG + Muscle Artifact	1.750	0.990	0.666	0.924	0.889	0.889 (0.879-0.899)	0.434
	Portable ECG + Muscle Artifact	2.000	0.990	0.672	0.923	0.890	0.890 (0.880-0.899)	0.437
	Portable ECG + Baseline Wander	0.500	0.993	0.560	0.956	0.880	0.880 (0.869-0.890)	0.402
	Portable ECG + Baseline Wander	0.750	0.992	0.607	0.943	0.885	0.885 (0.876-0.895)	0.419
	Portable ECG + Baseline Wander	1.000	0.992	0.630	0.942	0.888	0.888 (0.879-0.898)	0.427
	Portable ECG + Baseline Wander	1.250	0.991	0.645	0.934	0.890	0.890 (0.880-0.899)	0.432
	Portable ECG + Baseline Wander	1.500	0.991	0.656	0.930	0.891	0.891 (0.881-0.901)	0.437
	Portable ECG + Baseline Wander	1.750	0.990	0.663	0.925	0.892	0.892 (0.882-0.901)	0.440
	Portable ECG + Baseline Wander	2.000	0.990	0.668	0.924	0.892	0.892 (0.882-0.902)	0.443
Standard	Portable ECG + Electrode Motion	0.500	0.094	0.994	0.150	0.991	0.774 (0.760-0.788)	0.226
	Portable ECG + Electrode Motion	0.750	0.108	0.993	0.278	0.978	0.806 (0.793-0.819)	0.257
	Portable ECG + Electrode Motion	1.000	0.121	0.991	0.370	0.964	0.822 (0.810-0.835)	0.278
	Portable ECG + Electrode Motion	1.250	0.131	0.990	0.434	0.951	0.832 (0.820-0.844)	0.293
	Portable ECG + Electrode Motion	1.500	0.142	0.991	0.484	0.951	0.840 (0.828-0.852)	0.306
	Portable ECG + Electrode Motion	1.750	0.149	0.991	0.518	0.946	0.846 (0.834-0.857)	0.318
	Portable ECG + Electrode Motion	2.000	0.155	0.990	0.542	0.939	0.850 (0.839-0.862)	0.327
	Portable ECG + Muscle Artifact	0.500	0.093	0.991	0.141	0.985	0.773 (0.758-0.787)	0.234
	Portable ECG + Muscle Artifact	0.750	0.109	0.992	0.288	0.973	0.808 (0.795-0.821)	0.267
	Portable ECG + Muscle Artifact	1.000	0.124	0.991	0.394	0.959	0.827 (0.814-0.839)	0.287
	Portable ECG + Muscle Artifact	1.250	0.137	0.991	0.464	0.952	0.838 (0.826-0.850)	0.302
	Portable ECG + Muscle Artifact	1.500	0.149	0.991	0.513	0.949	0.845 (0.833-0.857)	0.315
	Portable ECG +Muscle Artifact	1.750	0.157	0.991	0.547	0.943	0.850 (0.838-0.861)	0.324
	Portable ECG + Muscle Artifact	2.000	0.164	0.990	0.573	0.935	0.854 (0.843-0.866)	0.331
	Portable ECG + Baseline Wander	0.500	0.096	0.992	0.171	0.984	0.783 (0.770-0.797)	0.218
	Portable ECG + Baseline Wander	0.750	0.119	0.994	0.351	0.975	0.816 (0.804-0.829)	0.254
	Portable ECG + Baseline Wander	1.000	0.136	0.992	0.455	0.960	0.832 (0.820-0.844)	0.277
	Portable ECG + Baseline Wander	1.250	0.150	0.991	0.519	0.947	0.842 (0.831-0.854)	0.293
	Portable ECG + Baseline Wander	1.500	0.159	0.990	0.557	0.935	0.849 (0.838-0.860)	0.305
	Portable ECG + Baseline Wander	1.750	0.167	0.990	0.585	0.931	0.854 (0.843-0.865)	0.315
	Portable ECG + Baseline Wander	2.000	0.174	0.989	0.605	0.928	0.858 (0.847-0.869)	0.325

SUPPLEMENTARY FIGURES

Figure S1. Flow chart of study cohort and analysis. Abbreviations: ECG, electrocardiogram; TTE, transthoracic echocardiogram.

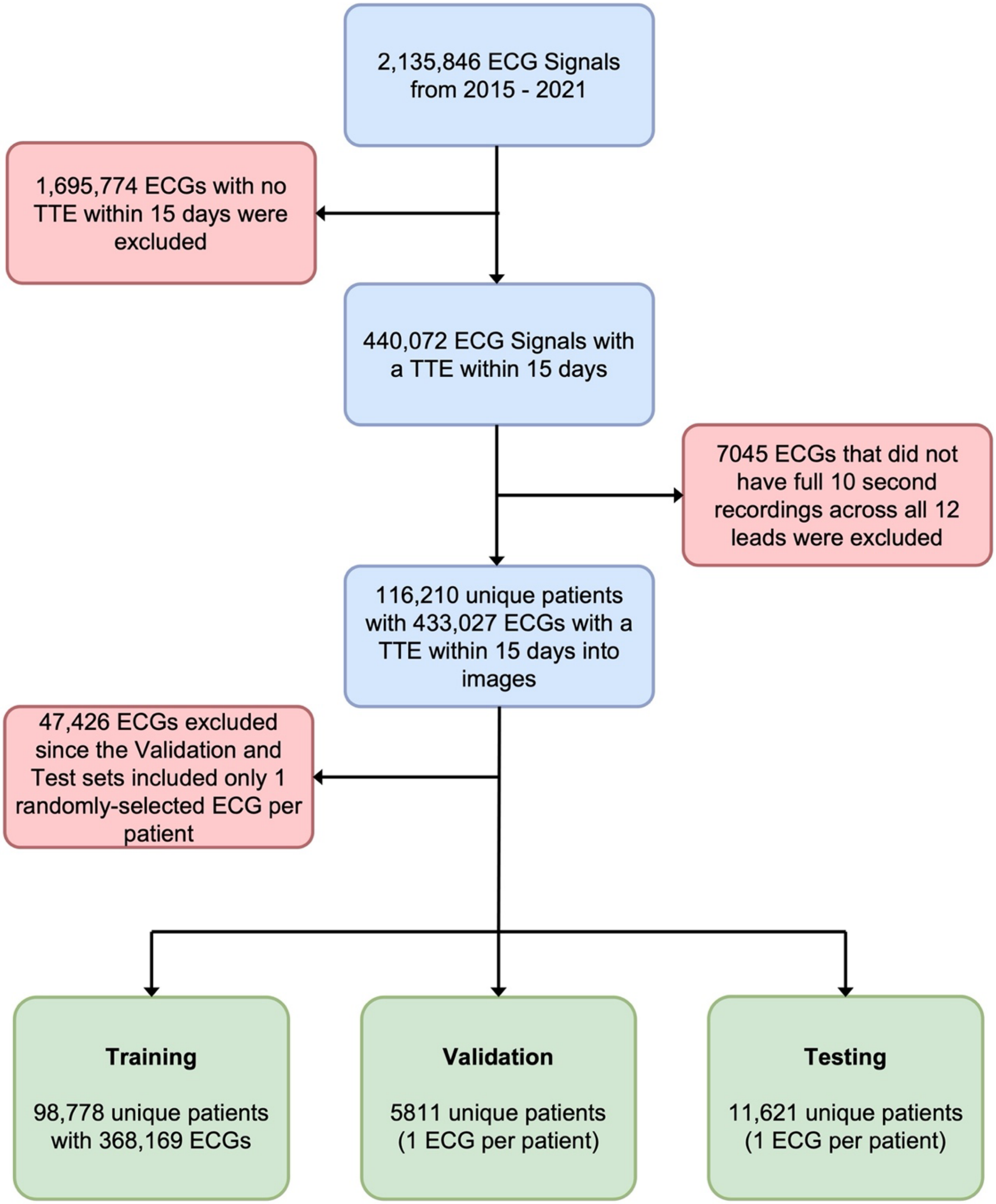

Figure S2. Model architecture used for model development. Abbreviations: ECG, electrocardiogram.

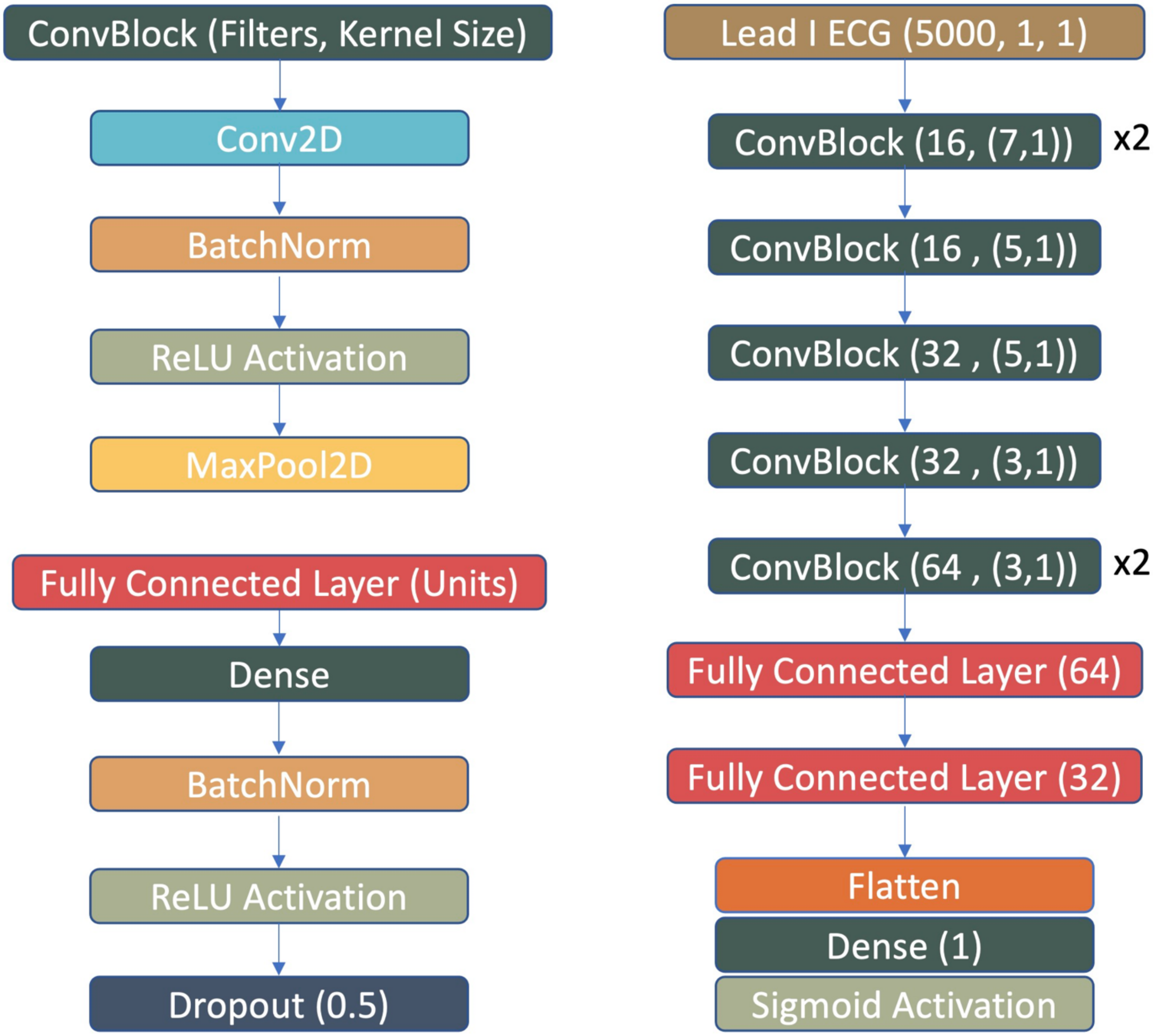

Supplement: Supplementary file 1 — Supplemental Material [file 41746_2023_869_MOESM1_ESM.pdf]
